# Prevalence and Associated Factors of Dual Physical and Cognitive Impairment Among Adults Aged 50 Years and Older with Cardiometabolic Conditions: A Cross-Sectional Study

**DOI:** 10.3390/healthcare14203451

**Published:** 2026-10-15

**Authors:** Kanokporn Pinyopornpanish, Suphawita Pliannuom, Peerasak Lerttrakarnnon, Sutina Laimangkorn, Pimchai Veerasirikul, Adchara Prommaban, Chaisiri Angkurawaranon

**Affiliations:** 1 Department of Family Medicine, Faculty of Medicine, Chiang Mai University, Chiang Mai 50200, Thailand; kanokporn.pinyopo@cmu.ac.th (K.P.); peerasak.lerttrakarn@cmu.ac.th (P.L.);; 2 Global Health and Chronic Conditions Research Group, Chiang Mai University, Chiang Mai 50200, Thailand; 3 Aging and Aging Palliative Care Research Cluster, Faculty of Medicine, Chiang Mai University, Chiang Mai 50200, Thailand

**Keywords:** sarcopenia, cognitive impairment, aging, pre-aging, non-communicable diseases

## Abstract

**Highlights:**

**What are the main findings?**

- Dual physical and cognitive impairment was identified in 25.00% of adults aged ≥50 years with NCDs attending a primary care clinic.
- Lower LDL cholesterol was significantly associated with a higher likelihood of dual physical and cognitive impairment, while older age and female sex showed potential associations.

**What are the implications of the main findings?**

- These findings highlight the substantial burden of dual impairment in primary care and support the importance of early screening and comprehensive assessment among adults with NCDs.

**Abstract:**

**Background:** Dual impairment of physical and cognitive capacities is associated with adverse health outcomes in aging populations and may emerge during middle age. However, evidence on its prevalence and associated factors among adults with cardiometabolic conditions in primary care settings remains limited, particularly in Southeast Asia. This study aimed to determine the prevalence of dual impairment and identify factors associated with dual impairment among adults aged ≥50 years with cardiometabolic conditions. **Methods:** A cross-sectional study was conducted among adults aged ≥50 years who had at least one cardiometabolic condition and attended a primary care clinic at a university-affiliated health center in Northern Thailand between March 2024 and July 2025. Dual impairment was defined as impaired cognitive function, assessed using the Montreal Cognitive Assessment (MoCA), together with impaired physical function, defined as either slow gait speed or low handgrip strength. Multivariable logistic regression analysis was used to examine factors associated with dual impairment. **Results**: A total of 196 participants were included. The mean age was 69.08 ± 6.15 years, and 72.96% were female. The prevalence of dual impairment was 25.00%. In the primary fully adjusted model (Model 1), lower LDL-C was the only factor significantly associated with dual impairment (adjusted OR [aOR] = 0.98, 95% CI: 0.97–1.00, *p* = 0.021). In the secondary parsimonious model (Model 2), female sex was associated with higher odds of dual impairment (aOR = 2.48, 95% CI: 1.05–5.84, *p* = 0.038), whereas age was not statistically significant (aOR = 1.04, 95% CI: 0.99–1.10, *p* = 0.139). The inverse association between LDL-C and dual impairment remained significant in Model 2 (aOR = 0.98, 95% CI: 0.97–0.99, *p* = 0.020). **Conclusions:** Dual impairment was identified in approximately one-quarter of participants, representing a substantial burden among individuals with cardiometabolic conditions. The observed inverse association between LDL-C and dual impairment should be interpreted cautiously given the potential for reverse causation and residual confounding, as well as the lack of information on lipid-lowering medication use. The identified associations are exploratory and warrant confirmation in larger prospective multicenter studies.

## 1. Introduction

Aging is closely associated with a gradual decline in multiple physiological functions, which can markedly affect independence, quality of life, and overall health outcomes, particularly among individuals with cardiometabolic conditions [1]. According to the World Health Organization’s (WHO) framework on Healthy Aging [2], declines in physical and cognitive capacities are key predictors of mortality and dependency in older adults [3]. Importantly, functional decline develops progressively across the life course rather than beginning abruptly in old age [4,5]. Cardiometabolic conditions, including hypertension, diabetes, dyslipidemia, and obesity, are common among middle-aged and older adults and have been associated with both physical and cognitive health [6,7]. Consequently, adults with cardiometabolic conditions may represent an important population for investigating the coexistence of physical and cognitive impairment before overt disability becomes clinically apparent.

On the physical side, one of the earliest indicators of declining functional capacity is the progressive reduction in muscle strength and muscle mass, which typically begins in middle age and accelerates with advancing age [4]. Although many studies have referred to frailty as a marker of impaired physical capacity, frailty is a broad and multidimensional construct that can be challenging to measure consistently [8]. In contrast, sarcopenia represents a more specific and objectively measurable phenotype of physical decline [9]. Physical decline in sarcopenia is characterized not only by reductions in muscle mass but also by decreased muscle strength and performance, impairments that compromise mobility and daily functioning. Sarcopenia is a complex physiological condition strongly associated with disability and an elevated risk of all-cause mortality [10,11,12,13,14]. Importantly, loss of muscle strength can occur even in the absence of measurable reductions in muscle mass and can independently predict functional decline and adverse health outcomes in older adults [15,16].

In parallel, cognitive function also declines with age. Cognitive changes can be detected as early as young adulthood [4,5], although different cognitive domains are affected at different rates. Mild cognitive impairment (MCI) represents a state of reduced cognitive capacity that is less severe than dementia, with daily functioning and independence generally preserved [17]. However, MCI can be progressive and may precede dementia in up to one-third of cases [18]. MCI has also been reported among individuals with cardiometabolic conditions, including diabetes and obesity, and may contribute to an increased vulnerability to cognitive decline [19].

Traditionally, declines in physical and cognitive capacities have been studied as separate domains. However, growing evidence suggests that they frequently co-occur [20,21] and may interact in ways that accelerate functional deterioration [22,23,24]. The co-occurrence of physical and cognitive impairment, referred to in this study as dual impairment, has gained increasing attention in geriatric research [25,26,27,28]. Individuals with dual impairment have been reported to be at increased risk of subsequent functional decline, dementia, dependence, and mortality compared with those with impairment in a single domain [26,27,29].

Several biological pathways have been proposed to explain the coexistence of physical and cognitive impairment, including vascular pathology, neuroinflammation, hormonal dysregulation, oxidative stress, chronic inflammation, and insulin resistance [30,31,32,33,34,35]. A better understanding of the prevalence of and factors associated with dual impairment may help inform future strategies for the early identification of individuals at risk. Such strategies may facilitate the development of integrated approaches targeting both physical and cognitive function to preserve functional independence and promote healthy aging.

Although physical and cognitive impairment have been extensively studied individually, evidence regarding their co-occurrence among middle-aged and older adults with cardiometabolic conditions in primary care remains limited, particularly in Southeast Asia. Therefore, the primary aim of this study was to determine the prevalence of dual impairment, and the secondary aim was to identify factors associated with its presence. Identifying these associated factors may contribute to hypothesis generation and inform future studies aimed at better characterizing and prospectively evaluating dual impairment in adults with cardiometabolic conditions.

## 2. Materials and Methods

### 2.1. Study Design and Setting

A cross-sectional study was carried out among middle-aged and older adults who had at least one cardiometabolic condition and attended a primary care outpatient clinic of the Department of Family Medicine at Chiang Mai University Hospital, a university-affiliated health center in Northern Thailand, between March 2024 and July 2025. The clinic primarily serves older adults. Based on previous research among patients with essential hypertension, who represent the largest patient population in this setting, approximately 60% of patients are women, with a mean age of 64.96 years (SD 9.04). Most patients were able to communicate in Thai, as assessed during the initial conversation when they were invited to participate in the study.

### 2.2. Participants

We recruited adults aged 50 years and older who regularly attended the primary care outpatient clinics and had at least one physician-diagnosed cardiometabolic condition documented in their medical records. The age threshold was selected to align with previous intrinsic capacity research [36]. Additionally, lifespan studies suggest that structural and functional brain changes may exhibit nonlinear trajectories and turning points during the fifth and sixth decades of life, with potential implications for cognitive functioning [37]. This age range therefore allowed us to examine physical and cognitive impairment across middle-aged and older adults with cardiometabolic conditions. We excluded individuals who were unable to perform the tests, including gait speed and hand grip strength assessments, those who had previously been diagnosed with dementia or depression, or those who were unable to communicate in Thai. Individuals with a previous diagnosis of dementia were excluded because the study aimed to evaluate cognitive impairment in participants without established dementia.

The required number of participants was determined using a single-proportion calculation, assuming an infinite population. The calculation assumed an expected prevalence (p) set at 0.15 [28], a margin of error (d) of 0.05, and a confidence level of 95%. Based on this calculation, the minimum required sample size was 196 participants.

### 2.3. Data Collection

Eligible patients were invited to take part in the study, and written informed consent was obtained before they were enrolled. A research assistant, independent of the clinical care team, approached participants individually while they were waiting for their medical consultation. Data collection was conducted on a single occasion for each participant.

Each participant completed a comprehensive health assessment, which included history taking, a review of medical records, a cognitive evaluation, and anthropometric measurements with body composition analysis. Participant characteristics were collected, including age, sex, education, comorbidities, and history of alcohol consumption and smoking. Cognitive function was measured using standardized cognitive tests, specifically the Thai version of the Montreal Cognitive Assessment (MoCA).

The physical examination involved measuring waist circumference (WC), blood pressure (BP), body weight, and height. Anthropometric parameters, including muscle mass and percentage of body fat, were obtained using a bioelectrical impedance analyzer (BIA; ACCUNIQ BC360; SELVAS Healthcare Inc., Seoul, Republic of Korea). This device has previously been validated against dual-energy X-ray absorptiometry (DXA) and showed a strong correlation for estimating lean body mass (*r* = 0.95), supporting its suitability for clinical research [38]. Muscle strength was evaluated with a handgrip dynamometer, whereas physical performance was measured by assessing usual gait speed [39].

### 2.4. Measures

#### 2.4.1. Physical Impairment

Skeletal muscle mass: Muscle mass was measured using bioimpedance analysis (BIA). Appendicular skeletal muscle mass (ASM) was calculated as the combined muscle mass of both arms and legs. The skeletal muscle index (SMI) was then calculated by dividing ASM by height in meters squared (kg/m^2^). Low muscle mass was defined as an SMI of <7.0 kg/m^2^ in men and <5.7 kg/m^2^ in women [40].

Muscle strength: Handgrip strength was measured using an analogue handgrip dynamometer (Takei 5001; Takei Scientific Instruments Co., Ltd., Tokyo, Japan) [41]. The dynamometer was calibrated before each use, and all measurements were performed by the same assessor. Participants completed two trials using their dominant hand while standing with the elbow fully extended, without a rest period. They received standardized verbal instructions to squeeze the dynamometer handle as firmly as possible for 3 s during each measurement. The best trial from two trials was used in the analysis [42]. Low grip strength was defined as <28 kg for men and <18 kg for women.

Physical performance: Physical performance was determined by usual gait speed, measured using the 6-m walking test [43]. Slow gait speed was defined as a walking speed of ≤0.8 m/s. This cutoff was selected based on previous evidence demonstrating its association with an increased risk of functional disability in older adults [43,44]. A sensitivity analysis using an alternative cutoff of <1.0 m/s was performed to assess the robustness of the findings.

Sarcopenia: Sarcopenia was defined according to the AWGS 2019 criteria as low skeletal muscle mass accompanied by either low muscle strength or low physical performance [9].

#### 2.4.2. Cognition Impairment

The Thai version of the MoCA was utilized to evaluate cognitive impairment, given its high validity in detecting MCI [45]. The maximum possible score is 30 points. To adjust for educational attainment, participants who had completed ≤6 years of schooling received an additional point. Cognitive impairment was defined as a Thai MoCA score of less than 25 [46], indicating MoCA-defined cognitive impairment rather than a clinical diagnosis of mild cognitive impairment.

#### 2.4.3. Dual Impairment

Dual impairment was defined as the presence of both cognitive and physical impairments in the same individual [28]. Participants were considered to have dual impairment if they met the criterion for cognitive impairment together with at least one indicator of physical impairment (slow gait speed and/or low handgrip strength).

### 2.5. Statistical Analysis

Statistical analyses were conducted using Stata version 16 (StataCorp LLC, College Station, TX, USA). Categorical data were reported as frequencies and percentages, whereas continuous variables were summarized as mean values with standard deviations (mean ± SD). Univariable associations between each factor and dual impairment were examined using Fisher’s exact test or Student’s *t*-test, as appropriate. The prevalence of physical impairment, including sarcopenia, low skeletal muscle index (SMI), low grip strength, and slow gait speed, was compared between participants with and without MoCA-defined cognitive impairment. Categorical variables were compared using Fisher’s exact test. These analyses were performed as exploratory unadjusted comparisons to describe the distribution of physical impairment according to cognitive status and were not intended to estimate independent associations. Univariable and multivariable logistic regression analyses were performed to identify factors associated with dual impairment. Crude and adjusted odds ratios (ORs) with 95% confidence intervals (CIs) were reported. Given the exploratory nature of the analysis, candidate variables were selected based on clinical relevance and the previous literature rather than solely on statistical significance in univariable analyses. Model 1 included all prespecified candidate variables: sex, age, body mass index (BMI), systolic blood pressure, fasting blood glucose, triglycerides, high-density lipoprotein cholesterol, low-density lipoprotein cholesterol, and alcohol use. Waist circumference and body fat percentage were excluded because they reflect overlapping aspects of adiposity. A parsimonious model (Model 2) was additionally fitted as a sensitivity analysis to assess the robustness of the findings and reduce model complexity. Model 2 included age, sex, BMI, fasting blood glucose, and low-density lipoprotein cholesterol. Sensitivity analyses were conducted by additionally adjusting for education and dyslipidemia, applying an alternative gait speed cutoff of <1.0 m/s, and performing subgroup analyses stratified by age (<65 vs. ≥65 years) to assess the robustness of the findings (Supplementary Materials). A VIF value of less than 5 was considered to indicate no meaningful multicollinearity. Model fit and influential observations were also assessed. A *p*-value < 0.05 was considered statistically significant.

## 3. Results

**Figure 1:**
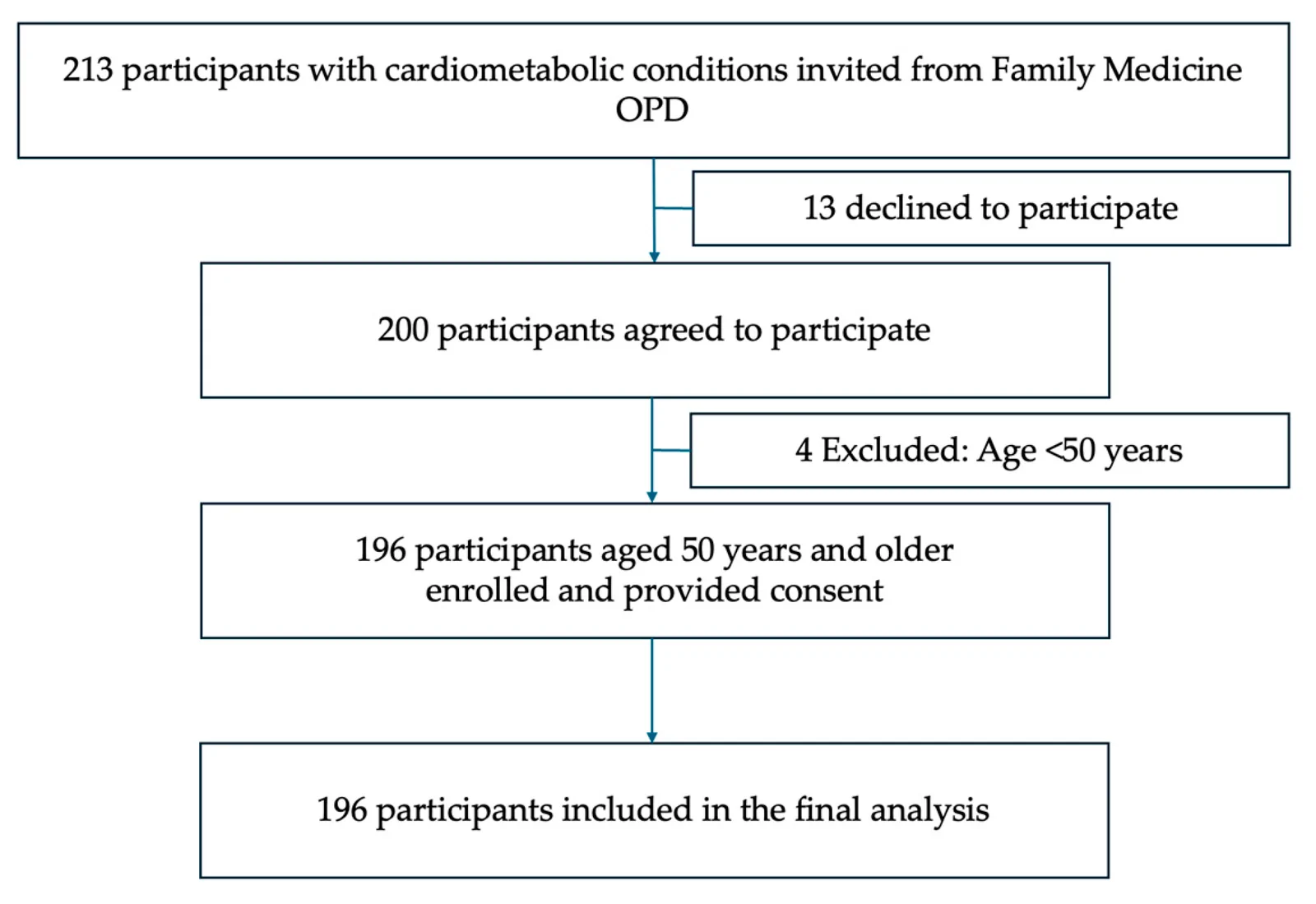
Study flow diagram.

A total of 196 participants aged 50 years and older were included in the analysis, of whom 49 (25.00%) had dual impairment, defined as the coexistence of physical and cognitive impairment (Figure 1). The mean age was 69.08 ± 6.15 years, and the study population was predominantly female (72.96%). Participants aged 50–64 years accounted for 20.41% of the study population (*n* = 40). The proportion of women was higher in the dual-impairment group than in the non-dual-impairment group (83.67% vs. 69.39%, *p* = 0.063). Regarding underlying diseases, dyslipidemia was present in 86.73% of participants. Participants with dual impairment had significantly lower LDL cholesterol levels than those without dual impairment (88.96 ± 23.05 vs. 97.14 ± 25.01 mg/dL, *p* = 0.045). No statistically significant between-group differences were observed in age or alcohol consumption (Table 1).

One hundred and twenty-three participants (62.75%) were classified as having MoCA-defined cognitive impairment. Regarding physical impairment, low skeletal muscle mass, low handgrip strength, and slow gait speed were present in 18.88%, 7.14%, and 36.22% of participants, respectively. Sarcopenia was identified in 13.27% of participants. Participants with MoCA-defined cognitive impairment had a significantly higher prevalence of low skeletal muscle mass than those without cognitive impairment (23.58% vs. 10.96%, *p* = 0.037). No statistically significant between-group differences were observed in low handgrip strength, slow gait speed, or sarcopenia (Table 2).

Multivariable logistic regression analysis showed that lower LDL cholesterol was significantly associated with higher odds of dual impairment in Model 1 (adjusted OR [aOR] = 0.98, 95% CI: 0.97–1.00, *p* = 0.021). In the parsimonious model (Model 2), female sex was significantly associated with higher odds of dual impairment (aOR = 2.48, 95% CI: 1.05–5.84, *p* = 0.038), whereas age was not statistically significant (aOR = 1.04, 95% CI: 0.99–1.10, *p* = 0.139). The association between lower LDL cholesterol and dual impairment remained statistically significant in Model 2 (aOR = 0.98, 95% CI: 0.97–0.99, *p* = 0.020). The association with female sex was model-dependent, reaching statistical significance only in Model 2 (Table 3). Sensitivity analyses showed that the association with female sex was attenuated after adjustment for education and dyslipidemia status (Supplementary Tables S1 and S2). The association between lower LDL cholesterol and dual impairment was no longer statistically significant when the alternative gait-speed cut-off was applied (Supplementary Table S3). Additional age-stratified analyses are presented in Supplementary Table S4.

## 4. Discussion

This study assessed the prevalence of dual impairment and explored factors associated with its presence among middle-aged and older adults with cardiometabolic conditions attending primary care clinics. In this study, the prevalence of dual impairment was around 26%. The prevalence of sarcopenia and MoCA-defined cognitive impairment was 9.5% and 62.5%, respectively. Exploratory multivariable analyses suggested possible associations of female sex, older age, and lower LDL cholesterol levels with dual impairment, although only lower LDL cholesterol remained statistically significant after adjustment.

The prevalence of dual impairment in our study was approximately one-quarter of participants. A study conducted at National Taiwan University Hospital used a definition of dual impairment comparable to that of the present study and reported a prevalence of 15.2% [28]. Notably, the Taiwanese study included an older population than our study, yet reported a lower prevalence. This difference may reflect variations in participant characteristics, study settings, and follow-up methods. In contrast, prior research on combined physical and cognitive impairments has mainly focused on cognitive frailty, generally defined as the presence of both physical frailty and cognitive impairment in individuals without dementia. For example, a multicenter study conducted in outpatient clinics in Bangkok, Thailand, reported a prevalence of cognitive pre-frailty and cognitive frailty of 14.4% and 6.7%, respectively [47], which was lower than that observed in our study. However, direct comparisons should be made cautiously because of differences in study populations and operational definitions. Further studies using standardized definitions are needed to better characterize the burden of dual impairment across different populations and clinical settings [48].

Studies have indicated that MoCA-defined cognitive impairment occurs more frequently among people with cardiometabolic conditions than in the general population [5]. Previous studies have estimated the prevalence of MCI to be approximately 10–20% among adults aged 65 years and older [49]. An elevated risk of MCI has been reported among individuals with metabolic syndrome (MetS) [50], and individuals with MCI also have an increased risk of progression to dementia [19]. In comparison with a survey from a primary care NCD clinic in India, which reported a prevalence of 12% (18.0% among older adults) [51], our study demonstrated a higher prevalence. However, it is broadly consistent with previous reports from populations with metabolic risk factors [52,53]. This relatively high prevalence may also reflect differences in participant characteristics, the inclusion of patients with chronic conditions, and the use of a screening instrument rather than a clinical diagnosis of MCI. Although the reasons for this high prevalence cannot be determined from our cross-sectional data, shared mechanisms proposed in previous studies include chronic inflammation, adiposity, insulin resistance, and vascular dysfunction associated with MetS, all of which have been proposed to contribute to cognitive impairment [54,55,56].

Compared with the pooled prevalence reported in a systematic review of the Thai population, our observed prevalence of sarcopenia was 13.27%. This prevalence was slightly lower than the pooled prevalence reported in a recent systematic review [57], but it was consistent with findings from outpatient clinic settings [58,59]. This difference may be explained by the characteristics of our study population, which included a majority of women, who generally have a lower prevalence of sarcopenia. In addition, we enrolled middle-aged participants, in whom sarcopenia is typically less common. Although sarcopenia tended to be more common among participants with MoCA-defined cognitive impairment, the difference was not statistically significant, consistent with previous studies [60,61].

Participants with MoCA-defined cognitive impairment had a higher prevalence of low skeletal muscle mass, whereas no clear differences were observed in muscle strength or gait speed. This pattern may reflect differences in what these measures capture, as muscle mass may be influenced by longer-term factors such as physical activity, nutritional status, metabolic health, and comorbidities [62], whereas muscle strength and gait speed are additionally influenced by neuromuscular function, balance, pain, and other aspects of overall physical function [63]. However, this comparison was descriptive and did not account for potential confounding. This finding should therefore be interpreted cautiously and explored further in future studies.

Interestingly, lower LDL cholesterol was associated with dual impairment in our exploratory multivariable analysis. However, this association was no longer statistically significant when an alternative gait-speed cut-off was applied in the sensitivity analysis. Although this relationship remains controversial, previous studies have reported associations between lower LDL cholesterol and cognitive impairment in patients with hypertension [64], as well as sarcopenia [65]. Given the cross-sectional design of this study, this finding should be interpreted with caution. Lower LDL cholesterol may reflect underlying frailty, malnutrition, or other health vulnerabilities rather than a direct risk factor for dual impairment. Nutritional vulnerability may also be closely related to functional health in older adults with chronic disease. For example, nutritional exposures have been associated with fatigue and activities of daily living in older adults with chronic disease [66]. This interpretation is supported by previous studies linking low LDL cholesterol with poor nutritional status [67] and frailty [68]. In older adults, low LDL cholesterol has been associated with frailty, occult illness, and poor nutritional status, raising the possibility of reverse causation and survivor bias [69]. In addition, residual confounding, including the absence of information on lipid-lowering medication use, may also have contributed to the observed association. Taken together, the attenuation of the LDL cholesterol association in the sensitivity analysis further supports cautious interpretation of this finding as an exploratory association rather than evidence of a causal relationship. Further longitudinal studies are needed to determine whether lower LDL cholesterol represents a marker of underlying vulnerability rather than a causal factor in dual impairment.

The lack of an association between BMI and dual impairment should also be interpreted cautiously. BMI is a useful and widely available measure of overall body size, but it does not distinguish between fat mass and lean mass and may therefore not adequately capture body composition and functional reserve in older adults. In particular, apparently normal or higher body weight may coexist with low muscle mass or declining muscle function. Recent evidence has emphasized the importance of preserving muscle mass and physical function during weight management in older adults and has highlighted the limitations of relying on BMI alone to characterize metabolic and functional health [70].

Older age and female sex showed potential associations with dual impairment in our exploratory analyses, although these findings were not consistent across models. These findings align with previous systematic reviews and meta-analyses of cognitive frailty, in which older age has been associated with a higher likelihood of coexisting physical and cognitive impairment [71], and older women have generally shown a higher prevalence [72]. More recent longitudinal population-based evidence from India has also reported higher odds of cognitive frailty among older women and those of more advanced age [73]. The higher prevalence of dual impairment with increasing age may reflect the accumulation of age-related changes affecting both physical and cognitive function. The mechanisms underlying the observed sex difference are likely multifactorial and may include differences in physical function, comorbidity, nutritional status, and social and socioeconomic factors. However, given the model-dependent findings and exploratory nature of our analyses, further studies in larger and more diverse populations are needed to determine whether age and sex are consistently associated with dual impairment.

The strength of this study lies in its focus on middle-aged and older adults attending outpatient clinics for cardiometabolic conditions, particularly those with metabolic risk factors, who face a higher likelihood of both physical and cognitive decline. In addition, we utilized standardized tools, including body impedance analysis, to assess physical capacity and identify dual impairment in the study population. This approach complements previous studies that relied primarily on clinical phenotypes rather than direct measurements of skeletal muscle mass. Although dual-energy X-ray absorptiometry (DXA) is considered the reference method for body composition assessment, BIA is more accessible, non-invasive, and feasible for routine use in primary care, particularly in resource-limited settings.

Several limitations should also be acknowledged. First, the cross-sectional design and single-center setting in northern Thailand preclude causal inference and may limit the generalizability of the findings. In particular, participants were recruited from a university-affiliated primary care clinic and may not fully represent community-dwelling adults or individuals receiving care in other primary care or clinical settings, who may differ in healthcare access, health status, and sociodemographic characteristics. Participants who were unable to communicate in Thai were excluded, which may have further limited the representativeness and external validity of the study population. This exclusion may have resulted in the underrepresentation of linguistically and culturally diverse populations. Second, cognitive function was assessed using the MoCA, a validated screening instrument that does not provide a comprehensive neuropsychological evaluation or establish a clinical diagnosis of MCI. Accordingly, the findings reflect MoCA-defined cognitive impairment rather than clinically diagnosed MCI. Direct comparisons with prevalence estimates from other studies should therefore be made cautiously because of potential differences in assessment methods and diagnostic criteria. Nevertheless, the MoCA is widely used for cognitive screening in research settings. Third, although handgrip strength measurements were performed by the same assessor using a calibrated dynamometer and standardized verbal instructions, the absence of a rest interval between the two trials may have introduced fatigue-related measurement error, potentially affecting the accuracy of grip strength measurements and the classification of low grip strength. Fourth, the study population consisted predominantly of women, which may partly reflect the patient profile of the study clinic [74,75] and may limit the applicability of the findings, particularly to male populations. In addition, although the rationale for including middle-aged adults was based on the potential for physical and cognitive changes to begin before old age, the relatively small number of participants in younger age groups limited the precision of age-specific estimates and the ability to conduct reliable subgroup analyses. Consequently, the findings should be interpreted cautiously when extrapolated across different age groups. Future studies involving larger populations with a broader age range and a more balanced sex distribution are warranted to further assess the generalizability of these findings. Fifth, although a parsimonious model was used for the exploratory regression analyses, the relatively modest sample size and the limited number of outcome events (n = 49) may have increased the risk of overfitting and resulted in imprecise or unstable estimates. The observed prevalence of dual impairment was lower than anticipated at the study design stage, resulting in fewer outcome events than expected and a lower events-per-variable ratio than initially anticipated in the primary model. Therefore, the regression findings should be interpreted cautiously as exploratory and hypothesis-generating. Finally, information on several potentially important confounders, including lipid-lowering medication use, physical activity, nutritional status, or depressive symptoms, was not available and may have resulted in residual confounding. Larger prospective multicenter studies with more comprehensive cognitive assessments and additional clinical information are needed to confirm these findings.

## 5. Conclusions

In conclusion, dual impairment was identified in approximately one-quarter of participants, suggesting that the coexistence of physical and cognitive impairment is not uncommon in this population. These findings provide preliminary evidence to support further investigation into integrated physical and cognitive assessment in primary care among adults aged 50 years and older with cardiometabolic conditions. The observed inverse association between LDL-C and dual impairment should be interpreted cautiously, as reverse causation and residual confounding cannot be excluded, and information on lipid-lowering medication use was unavailable. The association between female sex and dual impairment was observed only in the secondary parsimonious model and should be interpreted as model-dependent and exploratory. The identified factors should be interpreted as exploratory findings and warrant confirmation in larger, prospective multicenter studies. These findings may also inform the design of future longitudinal studies.

## Figures and Tables

**Table 1:** Demographic data of study participants.

Characteristic	Total (N = 196)	Dual Impairment		*p*-Value
		Yes (n = 49)	No (n = 147)	
Female gender, n (%)	143 (72.96)	41 (83.67)	102 (69.39)	0.063
Age, years (mean ± SD)	69.08 ± 6.15	70.18 ± 5.32	68.71 ± 6.38	0.148
Underlying diseases, n (%)				
- Dyslipidemia	170 (86.73)	39 (79.59)	131 (89.12)	0.095
- Hypertension	151 (77.04)	41 (83.67)	110 (74.83)	0.242
- Diabetes mellitus	53 (27.04)	14 (28.57)	39 (26.53)	0.853
Alcohol drinker, n (%)	30 (15.31)	4 (8.16)	26 (17.69)	0.167
Smoker, n (%)	1 (0.51)	0 (0)	1 (0.68)	1.000
Systolic BP, mmHg (mean ± SD)	132.30 ± 12.37	131.78 ± 12.51	132.48 ± 12.37	0.732
Diastolic BP, mmHg (mean ± SD)	73.30 ± 9.67	73.31 ± 9.50	73.31 ± 9.76	1.000
WC, cm (mean ± SD)	88.32 ± 10.77	86.82 ± 11.89	88.82 ± 10.37	0.260
BMI, kg/m^2^ (mean ± SD)	26.36 ± 3.87	26.07 ± 4.13	26.45 ± 3.79	0.550
Body fat percentage, % (mean ± SD)	34.19 ± 7.78	35.20 ± 8.48	33.86 ± 7.54	0.298
FBS, mg/dL (mean ± SD)	107.76 ± 21.07	106.12 ±21.44	108.31 ± 20.99	0.531
TG, mg/dL (mean ± SD)	114.36 ± 61.64	109.43 ± 44.13	116.01 ± 66.50	0.519
HDL cholesterol, mg/dL (mean ± SD)	60.25 ± 15.17	61.37 ± 15.77	59.87 ± 15.01	0.551
LDL cholesterol, mg/dL (mean ± SD)	95.09 ± 24.73	88.96 ± 23.05	97.14 ± 25.01	0.045

Abbreviations: BP = blood pressure; BMI = body mass index; FBS = fasting blood sugar; HDL = high-density lipoprotein; LDL = low-density lipoprotein; WC = waist circumference; SMI = skeletal mass index; TG = triglyceride.

**Table 2:** Distribution of physical parameters among participants with and without MoCA-defined cognitive impairment.

Physical Parameters	Total N = 196	CI N = 123	No CI N = 73	*p*-Value
Low SMI	37 (18.88)	29 (23.58)	8 (10.96)	0.037
Low grip strength	14 (7.14)	11 (8.94)	3 (4.11)	0.260
Low gait speed	71 (36.22)	46 (37.40)	25 (34.25)	0.759
Sarcopenia	26 (13.27)	19 (15.45)	7 (9.59)	0.282

Abbreviations: SMI = skeletal mass index; CI = cognitive impairment.

**Table 3:** Factors associated with dual impairment.

	Crude ORs	*p*-Value	Adjusted ORs (Model 1)	*p*-Value	Adjusted ORs of (Model 2)	*p*-Value
Female gender	2.26 (0.98 to 5.21)	0.055	2.21 (0.80 to 6.09)	0.124	2.48 (1.05 to 5.84)	0.038
Age, years *	1.04 (0.99 to 1.10)	0.149	1.04 (0.98 t o1.11)	0.164	1.04 (0.99 to 1.10)	0.139
BMI, kg/m^2^ *	0.97 (0.89 to 1.06)	0.548	0.99 (0.90 to 1.09)	0.861	0.99 (0.90 to 1.08)	0.812
Systolic BP, mmHg *	0.99 (0.97 to 1.02)	0.731	1.00 (0.97 to 1.02)	0.778	Not included	
FBS, mg/dL *	1.00 (0.98 to 1.01)	0.530	0.99 (0.97 to 1.01)	0.322	0.99 (0.97 to 1.01)	0.306
TG, mg/dL *	1.00 (0.99 to 1.00)	0.518	1.00 (0.99 to 1.01)	0.807	Not included	
HDL cholesterol, mg/dL *	1.01 (0.99 to 1.03)	0.549	1.00 (0.98 to 1.03)	0.858	Not included	
LDL cholesterol, mg/dL *	0.99 (0.97 to 1.00)	0.047	0.98 (0.97 to 1.00)	0.021	0.98 (0.97 to 0.99)	0.020
Alcohol drinker	0.41 (0.13 to 1.25)	0.118	0.69 (0.17 to 2.57)	0.582	Not included	

* Odds ratios (ORs) for continuous variables represent the change in odds associated with a one-unit increase. Reference categories were male for sex and non-drinkers for alcohol use. Abbreviations: BP = blood pressure; BMI = body mass index; FBS = fasting blood sugar; HDL = high-density lipoprotein; LDL = low-density lipoprotein; TG = triglyceride.

## Data Availability

The datasets used and/or analyzed during the current study are available from the corresponding author on reasonable request (contact S.P).

## Supplementary Materials

The following supporting information can be downloaded at: https://www.mdpi.com/article/10.3390/healthcare14203451/s1, Figure S1: Factors associated with dual impairment; Table S1: Multivariable logistic regression analysis of factors associated with dual impairment, additionally adjusted for formal education; Table S2: Multivariable logistic regression analysis of factors associated with dual impairment, additionally adjusted for formal education and dyslipidemia; Table S3: Sensitivity analysis using an alternative gait speed cutoff <1.0 m/s; Table S4: Age-stratified subgroup analyses.

## Abbreviations

The following abbreviations are used in this manuscript:

MCI	Mild cognitive impairment
MoCA	The Montreal Cognitive Assessment

## References

[1] Zhang K., Ma Y., Luo Y., Song Y., Xiong G., Ma Y., Sun X., Kan C. (2023). Metabolic diseases and healthy aging: Identifying environmental and behavioral risk factors and promoting public health. Front. Public Health.

[2] WHO (2017). Guidelines Approved by the Guidelines Review Committee. Integrated Care for Older People: Guidelines on Community-Level Interventions to Manage Declines in Intrinsic Capacity.

[3] At J., Bryce R., Prina M., Acosta D., Ferri C.P., Guerra M., Huang Y., Rodriguez J.J., Salas A., Sosa A.L. (2015). Frailty and the prediction of dependence and mortality in low- and middle-income countries: A 10/66 population-based cohort study. BMC Med..

[4] Hartshorne J.K., Germine L.T. (2015). When does cognitive functioning peak? The asynchronous rise and fall of different cognitive abilities across the life span. Psychol. Sci..

[5] Pinyopornpanish K., Buawangpong N., Soontornpun A., Thaikla K., Pateekhum C., Nantsupawat N., Wiwatkunupakarn N., Jiraporncharoen W., Angkurawaranon C. (2024). A household survey of the prevalence of subjective cognitive decline and mild cognitive impairment among urban community-dwelling adults aged 30 to 65. Sci. Rep..

[6] Koivunen K., Schaap L.A., Hoogendijk E.O., Schoonmade L.J., Huisman M., van Schoor N.M. (2022). Exploring the conceptual framework and measurement model of intrinsic capacity defined by the World Health Organization: A scoping review. Ageing Res. Rev..

[7] Aragoni da Silva J., Rolland Y., Martinez L.O., de Souto Barreto P. (2023). Mitochondrial Dysfunction and Intrinsic Capacity: Insights From a Narrative Review. J. Gerontol. A Biol. Sci. Med. Sci..

[8] Clegg A., Young J., Iliffe S., Rikkert M.O., Rockwood K. (2013). Frailty in elderly people. Lancet.

[9] Chen L.K., Woo J., Assantachai P., Auyeung T.W., Chou M.Y., Iijima K., Jang H.C., Kang L., Kim M., Kim S. (2020). Asian Working Group for Sarcopenia: 2019 Consensus Update on Sarcopenia Diagnosis and Treatment. J. Am. Med. Dir. Assoc..

[10] Lee J., Hong Y.P., Shin H.J., Lee W. (2016). Associations of Sarcopenia and Sarcopenic Obesity With Metabolic Syndrome Considering Both Muscle Mass and Muscle Strength. J. Prev. Med. Public Health.

[11] Donini L.M., Busetto L., Bischoff S.C., Cederholm T., Ballesteros-Pomar M.D., Batsis J.A., Bauer J.M., Boirie Y., Cruz-Jentoft A.J., Dicker D. (2022). Definition and Diagnostic Criteria for Sarcopenic Obesity: ESPEN and EASO Consensus Statement. Obes. Facts.

[12] Benz E., Pinel A., Guillet C., Capel F., Pereira B., De Antonio M., Pouget M., Cruz-Jentoft A.J., Eglseer D., Topinkova E. (2024). Sarcopenia and Sarcopenic Obesity and Mortality Among Older People. JAMA Netw. Open.

[13] Kim D., Lee J., Park R., Oh C.M., Moon S. (2024). Association of low muscle mass and obesity with increased all-cause and cardiovascular disease mortality in US adults. J. Cachexia Sarcopenia Muscle.

[14] Eitmann S., Matrai P., Hegyi P., Balasko M., Eross B., Dorogi K., Petervari E. (2024). Obesity paradox in older sarcopenic adults―A delay in aging: A systematic review and meta-analysis. Ageing Res. Rev..

[15] Clark B.C., Manini T.M. (2012). What is dynapenia?. Nutrition.

[16] Silva R.R., Galvão L.L., Meneguci J., Santos D.d.A.T., Virtuoso Júnior J.S., Tribess S. (2022). Dynapenia in all-cause mortality and its relationship with sedentary behavior in community-dwelling older adults. Sports Med. Health Sci..

[17] Bai W., Chen P., Cai H., Zhang Q., Su Z., Cheung T., Jackson T., Sha S., Xiang Y.-T. (2022). Worldwide prevalence of mild cognitive impairment among community dwellers aged 50 years and older: A meta-analysis and systematic review of epidemiology studies. Age Ageing.

[18] Lee J. (2023). Mild Cognitive Impairment in Relation to Alzheimer’s Disease: An Investigation of Principles, Classifications, Ethics, and Problems. Neuroethics.

[19] Ng T.P., Feng L., Nyunt M.S., Feng L., Gao Q., Lim M.L., Collinson S.L., Chong M.S., Lim W.S., Lee T.S. (2016). Metabolic Syndrome and the Risk of Mild Cognitive Impairment and Progression to Dementia: Follow-up of the Singapore Longitudinal Ageing Study Cohort. JAMA Neurol..

[20] Tolea M.I., Galvin J.E. (2015). Sarcopenia and impairment in cognitive and physical performance. Clin. Interv. Aging.

[21] Tessier A.-J., Wing S.S., Rahme E., Morais J.A., Chevalier S. (2022). Association of Low Muscle Mass With Cognitive Function During a 3-Year Follow-up Among Adults Aged 65 to 86 Years in the Canadian Longitudinal Study on Aging. JAMA Netw. Open.

[22] Grande G., Triolo F., Nuara A., Welmer A.K., Fratiglioni L., Vetrano D.L. (2019). Measuring gait speed to better identify prodromal dementia. Exp. Gerontol..

[23] Verghese J., Annweiler C., Ayers E., Barzilai N., Beauchet O., Bennett D.A., Bridenbaugh S.A., Buchman A.S., Callisaya M.L., Camicioli R. (2014). Motoric cognitive risk syndrome: Multicountry prevalence and dementia risk. Neurology.

[24] Montero-Odasso M., Almeida Q.J., Bherer L., Burhan A.M., Camicioli R., Doyon J., Fraser S., Muir-Hunter S., Li K.Z.H., Liu-Ambrose T. (2019). Consensus on Shared Measures of Mobility and Cognition: From the Canadian Consortium on Neurodegeneration in Aging (CCNA). J. Gerontol. A Biol. Sci. Med. Sci..

[25] Shimada H., Makizako H., Doi T., Park H., Tsutsumimoto K., Verghese J., Suzuki T. (2018). Effects of combined physical and cognitive exercises on cognition and mobility in patients with mild cognitive impairment: A randomized clinical trial. J. Am. Med. Dir. Assoc..

[26] Liu L.K., Chen C.H., Lee W.J., Wu Y.H., Hwang A.C., Lin M.H., Shimada H., Peng L.N., Loh C.H., Arai H. (2018). Cognitive Frailty and Its Association with All-Cause Mortality Among Community-Dwelling Older Adults in Taiwan: Results from I-Lan Longitudinal Aging Study. Rejuvenation Res..

[27] Einstad M.S., Schellhorn T., Thingstad P., Lydersen S., Aamodt E.B., Beyer M.K., Saltvedt I., Askim T. (2022). Neuroimaging markers of dual impairment in cognition and physical performance following stroke: The Nor-COAST study. Front. Aging Neurosci..

[28] Peng T.-C., Chen Y.-C., Chen T.-F., Chiou J.-M., Chen J.-H. (2021). Association of dual impairment in physical performance and cognition with cognitive change over two years in older adults. Alzheimer’s Dement..

[29] Tian Q., Montero-Odasso M., Buchman A.S., Mielke M.M., Espinoza S., DeCarli C.S., Newman A.B., Kritchevsky S.B., Rebok G.W., Resnick S.M. (2023). Dual cognitive and mobility impairments and future dementia—Setting a research agenda. Alzheimers Dement..

[30] Wiedmer P., Jung T., Castro J.P., Pomatto L.C.D., Sun P.Y., Davies K.J.A., Grune T. (2021). Sarcopenia—Molecular mechanisms and open questions. Ageing Res. Rev..

[31] Saedi E., Gheini M.R., Faiz F., Arami M.A. (2016). Diabetes mellitus and cognitive impairments. World J. Diabetes.

[32] van den Berg E., Reijmer Y.D., Biessels G.J., Biessels G.J., Luchsinger J.A. (2010). Cognition in Type 2 Diabetes or Pre-diabetic Stages. Diabetes and the Brain.

[33] Someya Y., Tamura Y., Kaga H., Sugimoto D., Kadowaki S., Suzuki R., Aoki S., Hattori N., Motoi Y., Shimada K. (2022). Sarcopenic obesity is associated with cognitive impairment in community-dwelling older adults: The Bunkyo Health Study. Clin. Nutr..

[34] Fakorede S., Lateef O.M., Garuba W.A., Akosile P.O., Okon D.A., Aborode A.T. (2025). Dual impact of neuroinflammation on cognitive and motor impairments in Alzheimer’s disease. J. Alzheimers Dis. Rep..

[35] Sargent L., Nalls M., Amella E.J., Slattum P.W., Mueller M., Bandinelli S., Tian Q., Swift-Scanlan T., Lageman S.K., Singleton A. (2020). Shared mechanisms for cognitive impairment and physical frailty: A model for complex systems. Alzheimers Dement..

[36] Chen M., Hanewald K., Si Y., Gu Y., Beard J.R. (2025). Intrinsic Capacity Across 15 Countries in the Survey of Health, Aging, and Retirement in Europe. JAMA Netw. Open.

[37] Dohm-Hansen S., English J.A., Lavelle A., Fitzsimons C.P., Lucassen P.J., Nolan Y.M. (2024). The ‘middle-aging’ brain. Trends Neurosci..

[38] Yang S.-W., Kim T.-H., Choi H.-M. (2018). The reproducibility and validity verification for body composition measuring devices using bioelectrical impedance analysis in Korean adults. J. Exerc. Rehabil..

[39] Han X., Zhang G.S., Li Q.R., Zhang Z. (2024). Current approach to the diagnosis of sarcopenia in cardiovascular diseases. Front. Nutr..

[40] Fukuoka Y., Narita T., Fujita H., Morii T., Sato T., Sassa M.H., Yamada Y. (2019). Importance of physical evaluation using skeletal muscle mass index and body fat percentage to prevent sarcopenia in elderly Japanese diabetes patients. J. Diabetes Investig..

[41] Yi D.W., Khang A.R., Lee H.W., Son S.M., Kang Y.H. (2018). Relative handgrip strength as a marker of metabolic syndrome: The Korea National Health and Nutrition Examination Survey (KNHANES) VI (2014–2015). Diabetes Metab. Syndr. Obes..

[42] Li Q., Cheng H., Cen W., Yang T., Tao S. (2024). Development and validation of a predictive model for the risk of sarcopenia in the older adults in China. Eur. J. Med. Res..

[43] Huang W.-F., Chen Y.-C., Chang C.-S., Wu C.-H. (2025). Definition and diagnosis of sarcopenia: Asia-Pacific perspectives. Osteoporos. Sarcopenia.

[44] de Souza A.F., de Oliveira D.C., Ramírez P.C., de Oliveira Máximo R., Luiz M.M., Delinocente M.L.B., Steptoe A., de Oliveira C., da Silva Alexandre T. (2025). Low gait speed is better than frailty and sarcopenia at identifying the risk of disability in older adults. Age Ageing.

[45] Nasreddine Z.S., Phillips N.A., Bédirian V., Charbonneau S., Whitehead V., Collin I., Cummings J.L., Chertkow H. (2005). The Montreal Cognitive Assessment, MoCA: A Brief Screening Tool For Mild Cognitive Impairment. J. Am. Geriatr. Soc..

[46] Hemrungrojn S., Tangwongchai S., Charoenboon T., Panasawat M., Supasitthumrong T., Chaipresertsud P., Maleevach P., Likitjaroen Y., Phanthumchinda K., Maes M. (2021). Use of the Montreal Cognitive Assessment Thai Version to Discriminate Amnestic Mild Cognitive Impairment from Alzheimer’s Disease and Healthy Controls: Machine Learning Results. Dement. Geriatr. Cogn. Disord..

[47] Wongtrakulruang P., Muangpaisan W., Panpradup B., Tawatwattananun A., Siribamrungwong M., Tomongkon S. (2020). The prevalence of cognitive frailty and pre-frailty among older people in Bangkok metropolitan area: A multicenter study of hospital-based outpatient clinics. J. Frailty Sarcopenia Falls.

[48] Ali N., Tian H., Thabane L., Ma J., Wu H., Zhong Q., Gao Y., Sun C., Zhu Y., Wang T. (2022). The Effects of Dual-Task Training on Cognitive and Physical Functions in Older Adults with Cognitive Impairment; A Systematic Review and Meta-Analysis. J. Prev. Alzheimers Dis..

[49] Langa K.M., Levine D.A. (2014). The diagnosis and management of mild cognitive impairment: A clinical review. JAMA.

[50] Wang X., Ji L., Tang Z., Ding G., Chen X., Lv J., Chen Y., Li D. (2021). The association of metabolic syndrome and cognitive impairment in Jidong of China: A cross-sectional study. BMC Endocr. Disord..

[51] Krishnamoorthy Y., Sarveswaran G., Sakthivel M., Rehman T., Majella M., Kumar S. (2019). Screening for mild cognitive impairment among noncommunicable disease patients attending a rural primary health center in Puducherry, South India. J. Nat. Sci. Biol. Med..

[52] Mehra A., Suri V., Kumari S., Avasthi A., Grover S. (2020). Association of mild cognitive impairment and metabolic syndrome in patients with hypertension. Asian J. Psychiatry.

[53] Zheng Y., Ma Q., Qi X., Zhu Z., Wu B. (2023). Prevalence and incidence of mild cognitive impairment in adults with diabetes in the United States. Diabetes Res. Clin. Pract..

[54] Assuncao N., Sudo F.K., Drummond C., de Felice F.G., Mattos P. (2018). Metabolic Syndrome and cognitive decline in the elderly: A systematic review. PLoS ONE.

[55] Amidfar M., Askari G., Kim Y.-K. (2024). Association of metabolic dysfunction with cognitive decline and Alzheimer’s disease: A review of metabolomic evidence. Prog. Neuro-Psychopharmacol. Biol. Psychiatry.

[56] Gulati K., Dubey H., Tanwar A., Arora A., Ray A., Gandhi T., Mehta A. (2024). Metabolic Syndrome and Associated Cognitive Dysfunction: A Pharmacological Perspective. Biochemical Mechanisms for Metabolic Syndrome.

[57] Whaikid P., Piaseu N. (2024). The prevalence and factors associated with sarcopenia in Thai older adults: A systematic review and meta-analysis. Int. J. Nurs. Sci..

[58] Therakomen V., Petchlorlian A., Lakananurak N. (2020). Prevalence and risk factors of primary sarcopenia in community-dwelling outpatient elderly: A cross-sectional study. Sci. Rep..

[59] Assantachai P., Phulsawat A., Ruengsinpinya P., Udompunturak S. (2021). Diagnostic accuracy of quadriceps strength-based criteria compared to handgrip-based criteria for diagnosing sarcopenia and severe sarcopenia in older adults. Arch. Gerontol. Geriatr..

[60] Peng T.C., Chen W.L., Wu L.W., Chang Y.W., Kao T.W. (2020). Sarcopenia and cognitive impairment: A systematic review and meta-analysis. Clin. Nutr..

[61] Batsis J.A., Haudenschild C., Roth R.M., Gooding T.L., Roderka M.N., Masterson T., Brand J., Lohman M.C., Mackenzie T.A. (2021). Incident Impaired Cognitive Function in Sarcopenic Obesity: Data From the National Health and Aging Trends Survey. J. Am. Med. Dir. Assoc..

[62] Tey S.L., Huynh D.T.T., Berde Y., Baggs G., How C.H., Low Y.L., Cheong M., Chow W.L., Tan N.C., Chew S.T.H. (2021). Prevalence of low muscle mass and associated factors in community-dwelling older adults in Singapore. Sci. Rep..

[63] Kim D.Y., Oh S.L. (2019). What is the Optimal Tool to Measure Gait Speed in a Clinical Setting?. Ann. Geriatr. Med. Res..

[64] Lv S., Jiao H., Zhong X., Qu Y., Zhang M., Wang R., Liu D., Yan X. (2025). Associations between serum lipid levels and cognitive impairment in hypertensive patients: A case-control study. BMC Neurol..

[65] Jiang Y., Xu B., Zhang K., Zhu W., Lian X., Xu Y., Chen Z., Liu L., Guo Z. (2023). The association of lipid metabolism and sarcopenia among older patients: A cross-sectional study. Sci. Rep..

[66] Arslan S., Bozkurt C., Arslan M., Bulut H. (2023). Effects of adherence to the Mediterranean diet on fatigue and activities of daily living in geriatric individuals with COPD. Clin. Nutr. ESPEN.

[67] Guligowska A., Stephenson S.S., Cieślak-Skubel A., Kravchenko G., Korycka-Błoch R., Kostka T.J., Chrzastek Z., Sołtysik B.K. (2023). Low total cholesterol levels are associated with a high risk of malnutrition in older adults. Clin. Nutr. ESPEN.

[68] Sato R., Matsuzawa Y., Yoshii T., Akiyama E., Konishi M., Nakahashi H., Minamimoto Y., Kimura Y., Okada K., Maejima N. (2024). Impact of Low-Density Lipoprotein Cholesterol Levels at Acute Coronary Syndrome Admission on Long-Term Clinical Outcomes. J. Atheroscler. Thromb..

[69] Zhou Z., Tonkin A.M., Curtis A.J., Murray A., Zhu C., Reid C.M., Williamson J.D., Ryan J., McNeil J.J., Beilin L.J. (2024). Low-Density-Lipoprotein Cholesterol and Mortality Outcomes Among Healthy Older Adults: A Post Hoc Analysis of ASPREE Trial. J. Gerontol. A Biol. Sci. Med. Sci..

[70] Arslan S., Aydın A. (2025). The weight-loss paradox in older adults: Balancing fat loss with muscle preservation. Bull. Natl. Res. Cent..

[71] Zhang T., Ren Y., Shen P., Jiang S., Yang Y., Wang Y., Li Z., Yang Y. (2021). Prevalence and Associated Risk Factors of Cognitive Frailty: A Systematic Review and Meta-Analysis. Front. Aging Neurosci..

[72] Qiu Y., Li G., Wang X., Zheng L., Wang C., Wang C., Chen L. (2022). Prevalence of cognitive frailty among community-dwelling older adults: A systematic review and meta-analysis. Int. J. Nurs. Stud..

[73] Sharma M., Anand A., Chattopadhyay A., Goswami I. (2024). Gender differentials in cognitive frailty among older adults in India: A multivariate decomposition approach. Sci. Rep..

[74] Thangsuk P., Pinyopornpanish K., Jiraporncharoen W., Buawangpong N., Angkurawaranon C. (2021). Is the Association between Herbal Use and Blood-Pressure Control Mediated by Medication Adherence? A Cross-Sectional Study in Primary Care. Int. J. Environ. Res. Public Health.

[75] Singhasiri P., Angkurawaranon C., Pliannuom S., Nantsupawat N., Pinyopornpanish K. (2025). Chronic pain in older adults with chronic diseases: Prevalence, perceived interference, and management strategies. BMC Geriatr..

