# Supplementary material for: Prevalence and Associated Factors of Dual Physical and Cognitive Impairment Among Adults Aged 50 Years and Older with Cardiometabolic Conditions: A Cross-Sectional Study
Source: Healthcare (Basel). 2026 Oct 15;14(20):3451. doi: 10.3390/healthcare14203451 (PMC13704316; doi:10.3390/healthcare14203451)
Supplement: Supplementary file 1 [file healthcare-14-03451-s001.zip › healthcare-4588152-supplementary.pdf]

SUPPLEMENTARY MATERIALS

Supplementary Results

Supplementary Table S1. Multivariable logistic regression analysis of factors associated with dual impairment, additionally adjusted for formal education.

Dual impairment	aOR	95% CI	p-value
Age, years	1.03	0.97-1.10	0.218
Female gender	1.71	0.69-4.24	0.243
BMI, kg/m^2	0.97	0.88-1.07	0.553
FBS, mg/dL	0.99	0.97-1.01	0.371
LDL cholesterol, mg/dL	0.98	0.97-1.00	0.046
Education	0.26	0.12-0.53	<0.001

Reference categories were male for sex and less than 6 years of education for education.

Abbreviations: BMI=body mass index; FBS= fasting blood sugar; LDL= low-density lipoprotein

Supplementary Table S2. Multivariable logistic regression analysis of factors associated with dual impairment, additionally adjusted for formal education and dyslipidemia.

Dual impairment	aOR	95% CI	p-value
Age, years	1.03	0.97-1.10	0.263
Female gender	1.85	0.74-4.65	0.189
BMI, kg/m^2	0.96	0.87-1.06	0.470
FBS, mg/dL	0.99	0.97-1.01	0.414
LDL cholesterol, mg/dL	0.98	0.97-1.00	0.038
Dyslipidemia	0.37	0.14-0.97	0.043
Education	0.25	0.12-0.53	<0.001

Reference categories were male for sex and less than 6 years of education for education.

Abbreviations: BMI=body mass index; FBS= fasting blood sugar; LDL= low-density lipoprotein

Supplementary Table S3. Sensitivity analysis using an alternative gait speed cutoff <1.0 m/s.

	Model 1			Model 2		
**Dual impairment**	aOR	95% CI	p-value	aOR	95% CI	p-value
Age, years	1.07	1.01-1.12	0.018	1.06	1.01-1.12	0.019
Female gender	3.80	1.55-9.32	0.004	3.58	1.71-7.47	0.001
BMI, kg/m^2	0.99	0.91-1.07	0.723	0.98	0.90-1.06	0.539
Systolic BP, mmHg	0.99	0.96-1.01	0.329		Not included	
FBS, mg/dL	1.00	0.98-1.02	0.926	1.00	0.98-1.01	0.920
TG, mg/dL	1.00	0.99-1.01	0.870		Not included	
HDL cholesterol, mg/dL	1.00	0.98-1.03	0.735		Not included	
LDL cholesterol, mg/dL	0.99	0.98-1.00	0.143	0.99	0.98-1.00	0.116
Alcohol drinker	1.01	0.35-3.18	0.927		Not included	

Reference categories were male for sex and non-drinkers for alcohol use.

Abbreviations: BP=blood pressure; BMI=body mass index; FBS= fasting blood sugar; HDL= high-density lipoprotein; LDL= low-density lipoprotein; TG= triglyceride

Supplementary Table S4. Age-stratified subgroup analyses

	Model 1			Model 2		
**Dual impairment**	aOR	95% CI	p-value	aOR	95% CI	p-value
**Age 50-64 (n=40)**						
Age, years	2.09	0.70-6.25	0.186	1.35	0.76-2.39	0.303
Female gender	Omitted			Omitted		
BMI, kg/m^2	0.50	0.23-1.08	0.078	0.65	0.37-1.13	0.125
Systolic BP, mmHg	1.00	0.91-1.33	0.308		Not included	
FBS, mg/dL	1.00	0.92-1.10	0.897	0.99	0.92-1.07	0.802
TG, mg/dL	0.99	0.95-1.03	0.755		Not included	
HDL cholesterol, mg/dL	0.95	0.84-1.07	0.380		Not included	
LDL cholesterol, mg/dL	0.93	0.86-1.00	0.052	0.93	0.86-1.00	0.052
Alcohol drinker	Omitted				Not included	
**Age ≥ 65 (n=156)**						
Age, years	1.01	0.93-1.11	0.734	1.02	0.93-1.11	0.709
Female gender	1.95	0.69-5.53	0.211	2.04	0.85-4.93	0.112
BMI, kg/m^2	1.03	0.93-1.14	0.579	1.03	0.93-1.13	0.619
Systolic BP, mmHg	1.00	0.97-1.03	0.784		Not included	
FBS, mg/dL	0.99	0.97-1.01	0.521	0.99	0.98-1.01	0.608
TG, mg/dL	1.00	1.00-1.01	0.524		Not included	
HDL cholesterol, mg/dL	1.01	0.98-1.03	0.687		Not included	
LDL cholesterol, mg/dL	0.99	0.97-1.00	0.129	0.99	0.97-1.00	0.143
Alcohol drinker	0.78	0.20-2.99	0.650		Not included	

Reference categories were male for sex and non-drinkers for alcohol use.

Abbreviations: BP=blood pressure; BMI=body mass index; FBS= fasting blood sugar; HDL= high-density lipoprotein; LDL= low-density lipoprotein; TG= triglyceride

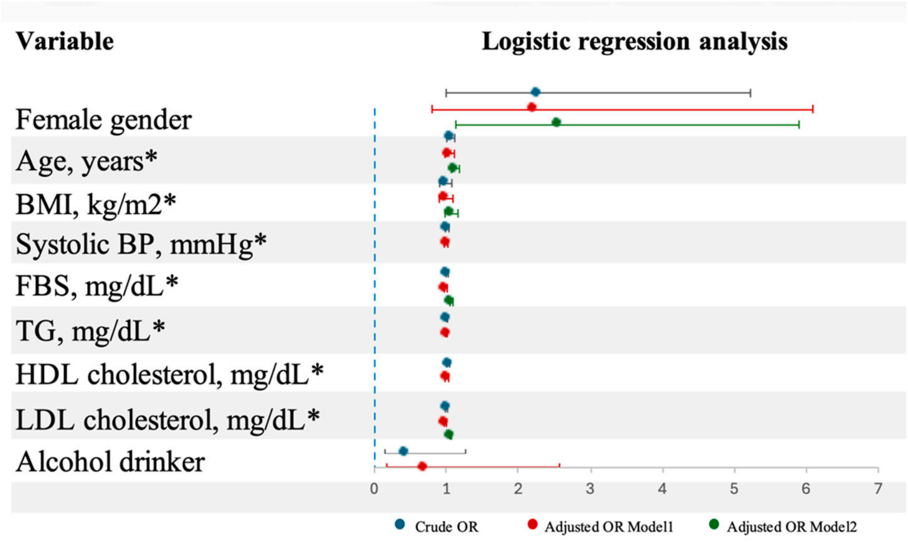

Supplementary Figure S1: Factors associated with dual impairment
